# Controller for microfluidic large-scale integration

**DOI:** 10.1016/j.ohx.2017.10.002

**Published:** 2017-10-31

**Authors:** Jonathan A. White, Aaron M. Streets

**Affiliations:** a Department of Bioengineering University of California, Berkeley, CA 94720, United States; b Chan Zuckerberg Biohub, San Francisco, CA 94158, United States

**Keywords:** Python, Arduino shield, Open source hardware, Solenoid valve, Microfluidics, Multi-layer soft lithography

## Abstract

Microfluidic devices with integrated valves provide precise, programmable fluid handling platforms for high-throughput biological or chemical assays. However, setting up the infrastructure to control such platforms often requires specific engineering expertise or expensive commercial solutions. To address these obstacles, we present a Kit for Arduino-based Transistor Array Actuation (KATARA), an open-source and low-cost Arduino-based controller that can drive 70 solenoid valves to pneumatically actuate integrated microfluidic valves. We include a python package with a GUI to control the KATARA from a personal computer. No programming experience is required.

Specifications Table.


Hardware nameKATARA Microfluidics Controller
Subject areaEngineering and Material Science; Biological SciencesHardware typeBiological sample handling and preparation; Electrical
Engineering and Computer ScienceOpen Source LicenseKATARA Software: MIT; KATARA Shield: Creative Commons
AttributionCost of Hardware∼$200 (Depends on throughput and
manufacturer)Source File Repositoryhttps://github.com/streetslab/KATARA-Microfluidics-Controller

## 1. Hardware in context

**Fig. 1:**
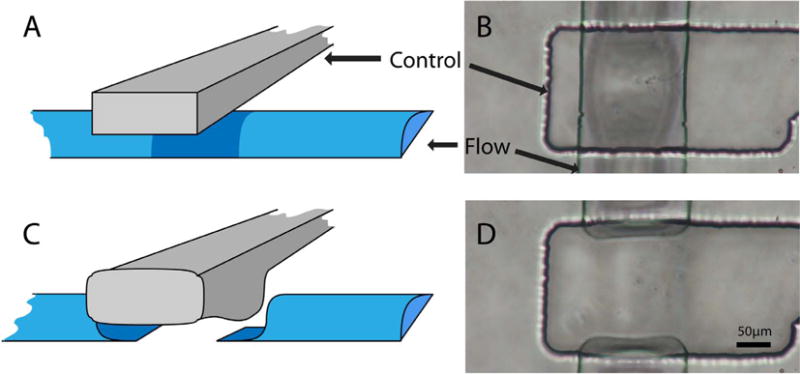
Cartoons and photographs of an integrated microfluidic valve in the open (A, B) and closed (C, D) position.

Microfluidic Large Scale Integration uses micromechanical valves integrated into silicone fluidic circuits to precisely and discretely manipulate picoliters to nanoliters of liquid. This allows scientists to perform quantitative biology experiments on microfluidic devices in parallel analogously to how integrated circuits use transistors to perform electronic computations in parallel [1–3]. Microfluidic devices with integrated valves have been developed for many applications including protein crystallization screens [4], single molecule conformation experiments [5], transcription factor binding affinity assays [6], cell culture assays [7,8], digital PCR [9], sandwich immunoassays [10,11], and single cell genomics [12,13]. A commonly used micromechanical valve, known as the Quake valve, uses pneumatically actuated control channels to pinch off adjacent flow channels by deforming the interstitial wall [1] (Fig. 1). This type of microfluidic valve can be manufactured in dense arrays using multilayer soft lithography [2,14]. A major barrier to entry for using microfluidic devices with integrated valves can be implementing control software and electronics. We aim to lower this barrier by introducing a Kit for Arduino-based Transistor Array Actuation (KATARA). The KATARA microfluidics control system is an open-source and low-cost platform for writing and running procedures that actuate solenoid valves.

## 2. Hardware description

**Fig. 2:**
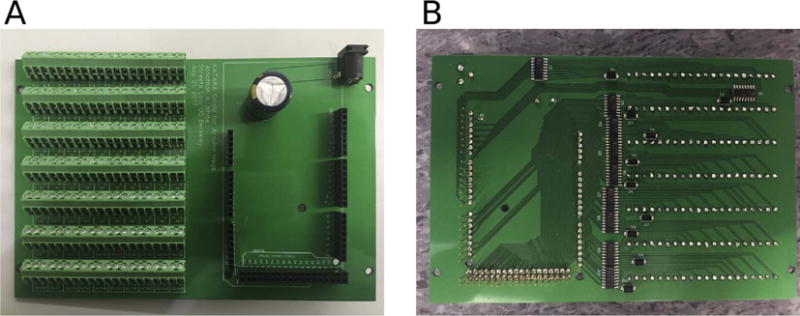
Photograph of the top (A) and bottom (B) side of an assembled KATARA shield. The top side of the KATARA shield has stackable headers, a power supply jack, and terminal blocks to attach solenoid valves. The bottom side has amplifying circuitry and the stackable header pins that plug into an Arduino Mega.

**Fig. 3:**
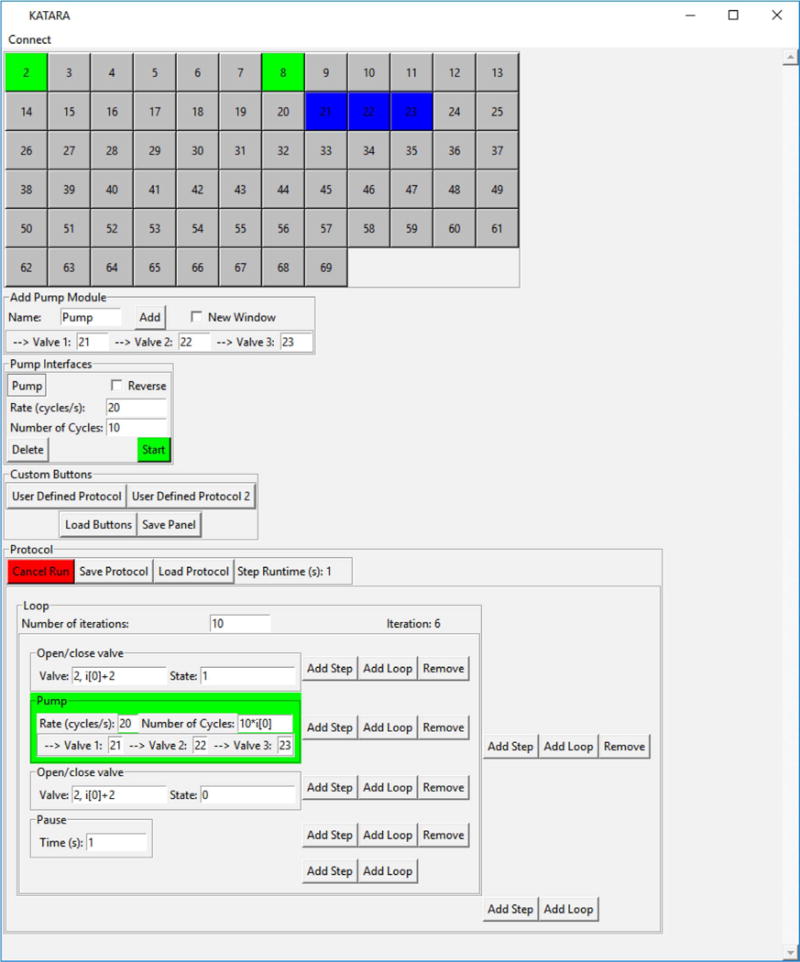
A screenshot of the KATARA GUI which has interfaces to actuate each individual valve; control peristaltic pumps; create, edit, run and save protocols; and load saved protocols as user defined buttons.

The KATARA microfluidics control system includes a shield for the Arduino Mega microcontroller that drives up to 70 solenoid valves (Fig. 2), Arduino firmware, and a python package with a graphical user interface (GUI) (Fig. 3). The package provides an interface to control Arduino-KATARA shield assemblies with python programs. The GUI allows users to actuate individual valves and three-valve peristaltic pumps [1] either manually or in any arbitrary automated sequence without any programming: users can create and edit protocols for pumping and actuating valves while iterating over loops. These protocols can be saved and loaded as custom buttons to build controls for any microfluidic device (see Supplementary Information). The KATARA python software and firmware use Arduino digital logic pins 0 and 1 to communicate, leaving 68 pins available to control valves. To use all 70 control lines on the shield, the Arduino must be programmed directly. Solenoid valve lead wires attach to (+) and (−) terminal pairs on the shield labeled 0–69 as they are referenced in the Arduino firmware and python software. Note that lines 54–69 are labeled A0-A15 on the Arduino (Fig. 2).

The KATARA shield is based on the open-source USB microfluidics controller designed by Rafael Gómez-Sjöberg [15], a circuit board that amplifies digital signals from a USB IO card to control 24 valves. By choosing surface-mount components, replacing the IO card with an Arduino, and streamlining valve connections, the KATARA shield can control nearly three times as many valves at a lower cost. In this issue, Brower et al. describe an alternative approach to control up to 48 valves and 18 sample inputs using a commercial Wago controller [16]. Another commercial device that can control microfluidic devices is the URMC32 digital relay from Numato. Additionally, other groups have described microcontroller-based control platforms [17,18,19]. The KATARA software can be extended to interface with these and other microfluidics controllers (see Supplementary Information).

Researchers who use the KATARA microfluidics control system will do so because it offers:

- A low-cost circuit to control up to 70 Solenoid valves,
- A python package that allows users to control the circuit board with python programs,
- A comprehensive GUI to write and share automated protocols for experiments: no programming experience is required.

## 3. Design files

**Table:** 

File	Description
KATARA_Shield_EASYEDA_Footprint.json	A source file for the KATARA shield footprint that is editable in EASYEDA
KATARA_Shield_EASYEDA_schematic_source.json	A source file for the KATARA shield schematic that is editable in EASYEDA
KATARA_Shield_Gerbers.zip	The gerber files for manufacturing the KATARA shield
Centroid_File.csv	The centroid file gives the locations of each component on the KATARA shield for assembly by pick and place machine
KATARA_Box_Power_USB_side.dwg	Autocad drawing of the KATARA containment box showing where to cut power jack, USB port, and terminal access holes
KATARA_Box_Power_USB_side.pdf	PDF drawing of the KATARA containment box showing where to cut power jack, USB port, and terminal access holes
KATARA_Box_terminal_side.dwg	Autocad drawing of the KATARA containment box showing where to cut a slit for solenoid valve wires
KATARA_Box_terminal_side.pdf	PDF drawing of the KATARA containment box showing where to cut a slit for solenoid valve wires
main.py	Main script to run the KATARA GUI
USB_GUI.py	Contains the base class for graphical user interfaces that connect to USB devices
KATARA_GUI.py	Contains classes for running the KATARA GUI and pump interfaces
Protocol_Tools.py	Contains classes for implementing protocol interfaces in the GUI
Step.py	Contains the base class for protocol steps
StepDerivatives.py	Contains derived step classes for pausing, pumping and actuating valves
no_wait_Dialog.py	Contains modified version of the Tkinter dialog window class that does not pause the running thread
LabelEntry.py	Contains a class for drawing and referencing labeled text entry bars
KATARA.ico	The KATARA icon
ValveController.py	Contains ValveController and peristalticPump classes (described in Supplementary Information section 4)
KATARAValveController.py	Contains the KATARAValveController and KATARAPump classes for sending serial signals to Arduinos running the KATARA firmware. These classes are the programming interface and are used internally in the GUI (see Supplementary Information section 3)
Config.py	Contains variables that GUI related classes in different files share
KATARA_Firmware.ino	The Arduino program that interprets and executes serial commands sent from the KATARAValveController and KATARAPump classes

## 4. Bill of materials

**Table:** 

Designator	Component	Number	Cost Per Unit	Total Cost	Supplier	Manufacturer Part Numb	Material type
Q1-Q10	Bipolar (BJT) Transistor Array 7 NPN Darlington 50 V 500 mA Surface Mount 16-SOIC	10	$0.45	$4.51	Digikey	MC1413BDG	Semiconductor
D1-D10	Zener Diode 22 V 1 W ± 5% Surface Mount SMA	10	$0.38	$3.84	Digikey	SMAZ22-13-F	Semiconductor
P1	Conn PWR Jack 2.5 × 5.5 MM Solder	1	$0.64	$0.64	Digikey	PJ-102B	Metal/Polymer
MP1-MP7	CONN TERM BLOCK 45DEG 10PS 3.5 MM	14	$1.99	$27.86	Digikey	1989036	Metal/Polymer
R1	RES SMD 10 K OHM 5% 1/16 W 0402	1	$0.10	$0.10	Digikey	RC0402JR-0710KL	Composite
C1	CAP ALUM 1000UF 20% 35 V RADIAL	1	$1.15	$1.15	Digikey	EEU-FC1V102S	Metal
U29	Arduino MEGA Stackable Header Kit	1	$1.50	$1.50	Itead	IM120531023	Metal/Polymer
	AC/DC DESKTOP ADAPTER 24 V 120 W	1	$96.88	$96.88	Digikey	PSA120U-240L6	Metal/Polymer/Semiconductor
	Arduino Mega 2560 Rev3	1	$45.95	$45.95	Arduino	A000067	Semiconductor
	Printed Circuit Boards	5	$4.98	$24.90	EasyEda	https://easyeda.com/jwhite2/Driver_Shield_for_A-rduino_Mega-479918a931264db-daa817511-dac3210f	Metal/Polymer
	Solder Stencil*	1	$13.00	$13.00	EasyEda	https://easyeda.com/jwhite2/Driver_Shield_for_A-rduino_Mega-479918a931264db-daa817511-dac3210f	Metal
	Solder Paste	1	$15.95	$15.95	Digikey	SMD291SNL	Metal
	BOX ABS BLACK 7.61“L X 4.61”W	1	$11.70	$11.70	Digikey	CU-3284	Polymer
	1/2 inch Spacers	10	$0.17	$1.66	Grainger	13SP057	Polymer
	M2.5 20 mm screws	100	$0.07	$6.65	Grainger	M51340.025.0020	Metal

† Components with reference designators should be included in the bill of materials submitted to PCB assemblers.

* These items are necessary for do-it-yourself assembly, but they will be included as part of commercial assembly services.

## 5. Validated solenoid valves

**Table:** 

Component	Number	Cost Per unit	Total Cost	Manufacturer	Part number	Material type
Pneumadyne 24 V solenoid valve	1–70	$25.18	$25.18–$1762.60	Pneumadyne	S10MM-31-24-2	Metal/Polymer
Pneumadyne Manifold	1–7	$28.09	$28.09–$196.63	Pneumadyne	MSV10-10	Metal
Nitra 24 V solenoid valve	1–70	$18.50	$18.50–$1295	Nitra Pneumatics	AVP-31	Metal/Polymer
Nitra Valve Stacking kit	0–69	$1.75	$0–$120.75	Nitra Pneumatics	AVP-31 KIT	Metal/Polymer

## 6. Build instructions

### 6.1. Hardware Design

**Fig. 4:**
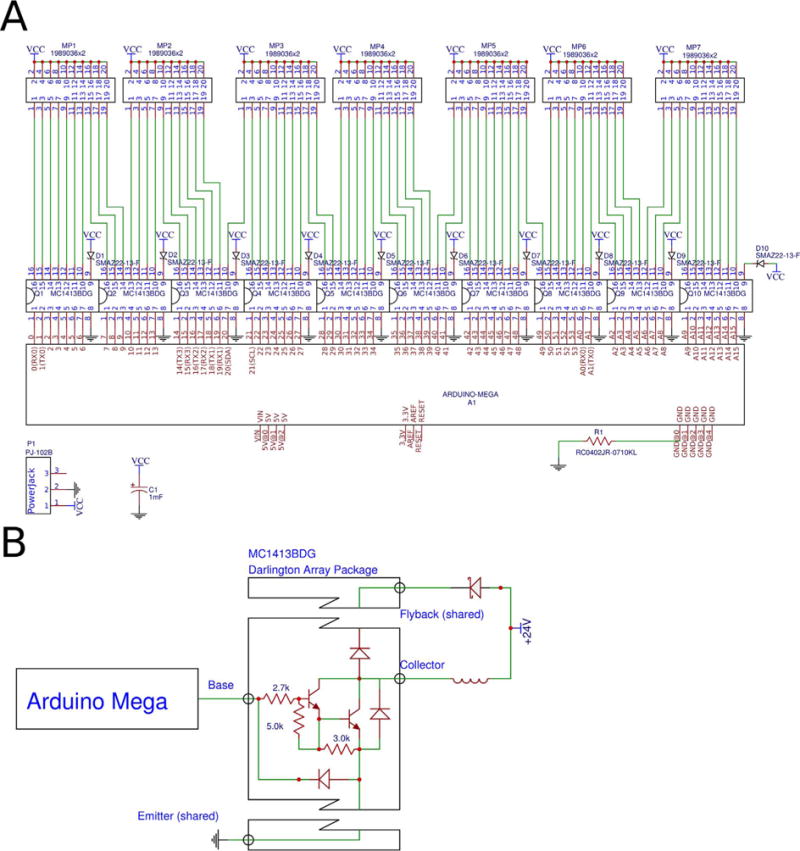
An electrical schematic of the whole KATARA shield (A) and of a single amplifying circuit on the KATARA shield (B).

The KATARA shield extends the Arduino Mega by amplifying signals from each of its digital pins to drive a solenoid valve. To do this, it uses ten array packages each containing seven Darlington pair amplifier circuits (Fig. 4). Darlington amplifiers offer no performance advantage over simple transistor amplifiers for our application, but Darlington array packages reduce the cost and number of components necessary to build the board. The Darlington arrays include a shared flyback pin that is connected to the source voltage across the cathode of a Zener diode with 22 V breakdown voltage (Fig. 4): placing Zener diodes here forces solenoid valves to close faster and ensures that the voltage across the amplifier will never exceed its 50 V rating when driving 24 V valves. The KATARA shield can also drive valves operating at voltages less than 24 V without modification. We successfully controlled 24 V Nitra AVP-31(Nitra Pneumatics) and 5 V LHDA0521111H (The Lee Company) valves using the KATARA (data not shown). If choosing valves other than the Pneumadyne S10MM-31-24-2 to use with the KATARA shield, choose a power supply at the valve’s operating voltage with a current rating sufficient to drive all attached valves and ensure that the average power dissipated through any valve does not exceed its maximum operating power. To ensure the power dissipated across the Zener diodes stays within specifications, limit the number of times any individual Darlington array package closes valves per second to

$$n_{\max}=\frac{2\mathrm{Watts}}{LI^{2}},$$

where *L* is the inductance of the valve and *I* is the current it conducts while energized. For the Pneumadyne S10MM-31-24-2, *n*_max_ is greater than 100,000, but exceeding *n*_max_ may be a concern with solenoid valves that draw more power. The KATARA shield also includes a 10 kΩ resistor, which connects the Arduino and external supply grounds to establish a reference for the control circuit while shielding the Arduino and the computer from current spikes, and a 1 mF capacitor, which ensures enough energy is always on hand to open solenoid valves quickly.

### 6.2. Hardware assembly

**Fig. 5:**
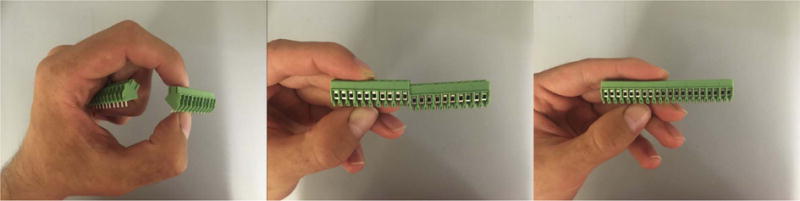
Demonstration of how to join terminals with interlocking sides.

**Fig. 6:**
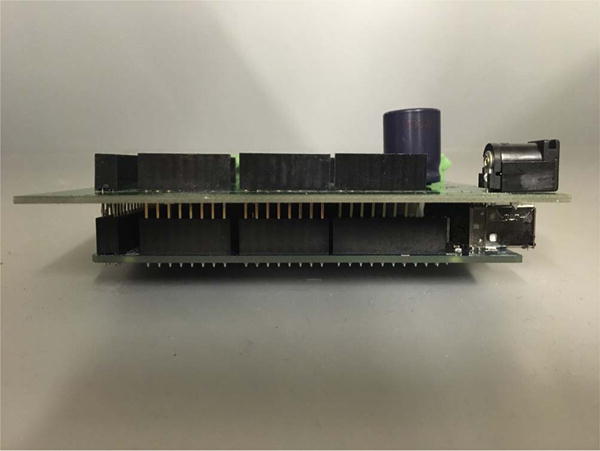
A KATARA shield plugged into an Arduino Mega (side view).

**Fig. 7:**
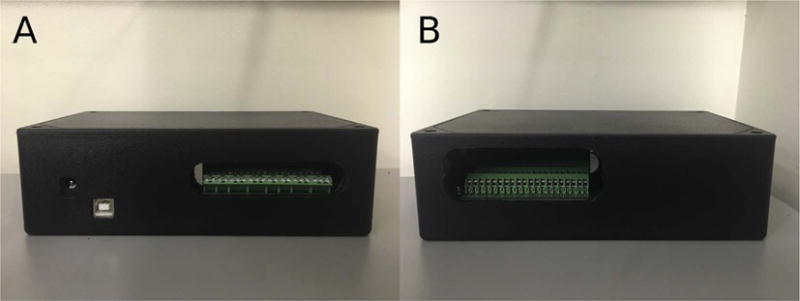
The KATARA shield inside a protective box. Holes give access to the power jack, USB port, and terminal screws on one side (A), and a slit provides an entryway for solenoid valve wires on the other side (B).

**Fig. 8:**
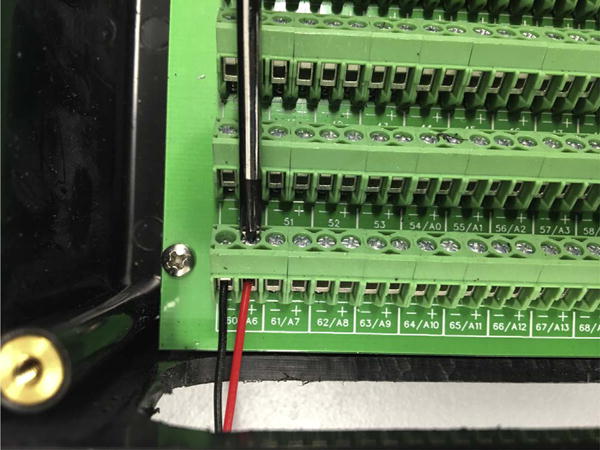
Solenoid valve lead wires are clamped into place by tightening the screws on the terminal blocks.

One may order assembled KATARA shields from PCB manufacturers by submitting the gerber files, centroid file, and bill of materials. To assemble the KATARA shield yourself, order an unassembled board and use the following procedure:

1. Apply solder paste to the surface mount pads on the bottom side of the board with the solder stencil.
  ◦ Reference [20] is a good tutorial on how to apply solder paste with solder stencils.
  ◦ If solder paste is accidentally applied between contact pads, surface tension will remove the connections during reflow when it pulls molten solder onto each pad.
2. Use tweezers to place the surface mount resistor (R1), transistor arrays (Q1-Q10), and Zener diodes (D1-D10) with the correct orientation.
  ◦ The white bar on the Darlington array should be oriented with the white circle in the corner of the array outline printed on the PCB.
  ◦ The white line printed on the diode should be oriented with the white line in the outline of the diode printed on the PCB.
3. Reflow solder:
  ◦ This can either be done with a reflow oven using the recommended heating profile [21] or by heating the board on a hot plate from room temperature until the solder melts at 220 °C [22] to approximate the recommended heating profile and limit heat shock to the components. Be sure to perform this step in a well ventilated area.
4. Remove any excess solder that might short adjacent pads with solder wick and a soldering iron.
5. Join seven pairs of ten-position Phoenix Contact block terminals with interlocking sides to produce seven twenty-position block terminals (Fig. 5).
6. Solder the terminal blocks and power jack on the top side of the board with a soldering iron and solder wire.
7. Solder the stackable Arduino Mega headers taking care to install them all perpendicular to the board: if they point at different angles from each other, the shield will be very difficult to plug into an Arduino.
  ◦ First, solder one pin of each header. Then align the shield to an Arduino Mega and adjust misaligned headers by reheating the single soldered joint. Make sure that the shield easily plugs into the Arduino before soldering the rest of the pins. See reference [23] for a full tutorial.
8. Solder the capacitor (take care to install with the correct polarity) and then clip the leads.
9. Plug the KATARA shield into an Arduino Mega (Fig. 6).
10. Cut holes in the electrical box (CU-3284) according to the KATARA_Box_terminal_side and KATARA_Box_USB_Power_side design files (Fig. 7).
11. Place the KATARA shield-Arduino assembly into the box. Fasten the assembly to the the box with four M2.5 20 mm screws and ½ inch spacers raising it off the box’s PCB screw pedestals.
12. Thread solenoid valve lead wires through the slit and attach them to the terminal blocks. Clamp the leads in place by tightening the screws when the board is not powered to avoid shocking hazard (Fig. 8).

## 7. Operation instructions

To operate the KATARA shield, first install the KATARA firmware to the Arduino using the Arduino IDE. The KATARA python software requires python 2.7 and the pyserial package. To run the GUI, open a terminal window and navigate into the KATARA_Software folder (available on Github), then run the command:


python main.py


Once the software is open and connected to the Arduino, the user may open and close valves manually, specify pump modules, edit and save protocols, and load saved protocols as custom control buttons. For more detailed instructions on how to use and install the KATARA Firmware and Software, see the Supplementary Information.

## 8. Hardware validation

**Fig. 9:**
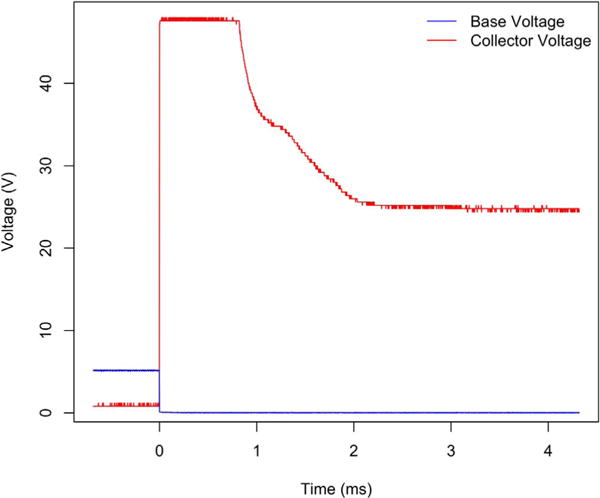
The electrical response of Pneumadyne S10MM-31-24-2 solenoid valves when the KATARA shield switches them off (at time zero) takes less than three milliseconds.

To evaluate the performance of the KATARA amplifier circuit, we measured the voltage between the collector of a Darlington amplifier and ground with an oscilloscope (TBS 1052B-EDU Tektronix) as the circuit switched off a Pneumadyne S10MM-31-24-2 solenoid valve (Fig. 9). Fig. 9 shows that before switching off at time zero, the digital output from the Arduino at the amplifier base is high and the voltage at the collector is zero. At time zero when the Arduino’s signal to the transistor base goes low, the amplifier circuit stops conducting. The voltage at the collector then spikes as the solenoid continues to drive current, but plateaus when the voltage across the Zener diode reaches its breakdown level. The high collector voltage reverses the current through the solenoid, then drops below the Zener breakdown level after about one millisecond and decays to the source voltage within another two milliseconds (Fig. 9). This demonstrates that the electrical response of the KATARA circuit connected to Pneumadyne S10MM-31-24-2 valves is under three milliseconds, which is less than the valves’ specified ten millisecond de-energization time and on the same order as a microfluidic valve’s response time [1]. The response time of integrated microfluidic valves depends on many additional factors including the tubing size, material, and length, and the PDMS stiffness which is determined by device fabrication protocols. To demonstrate that the circuit is capable of controlling a microfluidic device with integrated pumps, we used the KATARA control system to drive a peristaltic pumping sequence (Supplementary Video 1).

**Fig. 10:**
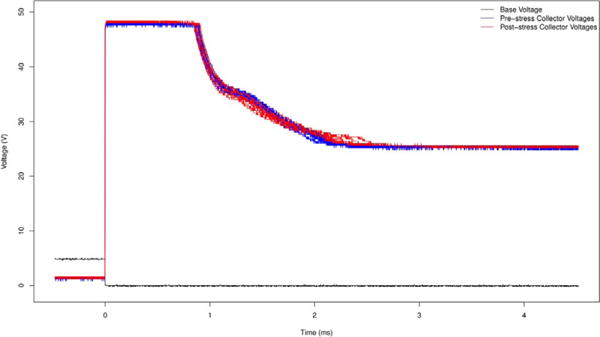
The electrical response when switching off fourteen Pneumadyne S10MM-31-24-2 solenoid valves before (blue) and after (red) running them continuously for a week.

To ensure that the transistors are robust to prolonged operation, we drove solenoid valves with every transistor on two Darlington arrays for a week. One of the two Darlington arrays drove all seven valves continuously while the other array drove four valves continuously and operated the other three in a peristaltic pumping cycle at 10 Hz. Measurements of the electrical response before and after the week long operation show essentially no change in the switching behavior of the circuit (Fig. 10). The inductive tail of the response lengthened slightly which may be due to wear on the solenoid valves.

## 9. Discussion

The KATARA provides a user-friendly solution to control solenoid valves at low cost. A complete microfluidic platform must also include the pneumatic infrastructure to relay pressure to the solenoid valves and operate the microfluidic device. In this issue, Brower et al. present a comprehensive pneumatic platform for microfluidic large-scale integration [16]. The KATARA may be used as an alternate control module in this platform, as it serves as a low-cost alternative to the Wago controller. The KATARA shield also has the capability to control microfluidic devices remotely without a computer. This opens up the possibility to use microfluidic devices with integrated valves in field settings. The KATARA shield also maintains the Arduino’s ability to use its digital pins for purposes other than driving solenoid valves when valves are not connected to their amplifying circuits. Additionally, the KATARA may be suitable for purposes other than microfluidics including soft robotics, driving motors, and powering light sources. As we continue to develop the KATARA, we will post hardware and software updates on the Streets Lab website (http://streetslab.berkeley.edu/tools/katara/).

## Supplementary Material

Supplemental Video

Supplementary information

## Appendix A. Supplementary data

Supplementary data associated with this article can be found, in the online version, at http://dx.doi.org/10.1016/j.ohx.2017.10.002.
