# Supplementary material for: Controller for microfluidic large-scale integration
Source: HardwareX. Author manuscript; Available in PMC 2019 Feb 13. (PMC6373447; doi:10.1016/j.ohx.2017.10.002)
Supplement: Supplementary information [file NIHMS975709-supplement-Supplementary_information.docx]

**Supplementary Information**

**Contents**

1. Installation Instructions
2. KATARA GUI Manual
3. KATARA Programming Documentation
4. Extending the KATARA API and GUI to interface with valve control devices other than the KATARA Shield

**1. Installation Instructions**

The KATARA GUI connects to an Arduino Mega running the KATARA firmware. To upload the KATARA firmware to an Arduino Mega, open the KATARA firmware source code with the Arduino IDE (<https://www.arduino.cc/en/Main/Software>). Make sure the correct board type is selected in the Arduino IDE’s dropdown menu under Tools>>Board and the correct port is selected under Tools>>Port. To compile the code and upload it to the board, click the arrow button at the upper left hand corner of the Arduino IDE. If the code does not compile, install a new version of the Arduino IDE. When finished, “Done Uploading” should be written at the top of the terminal.

The KATARA GUI runs with python 2.7 which comes preinstalled on macs and linux. There are many ways to install python 2.7 on windows, but we use the Enthought Canopy distribution. To run the KATARA software, you will first need to install the pyserial package which is available through both pip and the Enthought Canopy package manager. After installing pyserial, download the KATARA Software from its Github repository (<https://github.com/jonathanawhite1381/KATARA-Microfluidics-Controller>) and it should be ready to run.

**2. KATARA GUI Manual**

To run the KATARA GUI, open a terminal window and navigate into the KATARA_Software folder (available on Github). Then type

python main.py

and press enter. The KATARA GUI should open and you will see its main window (Figure S1).


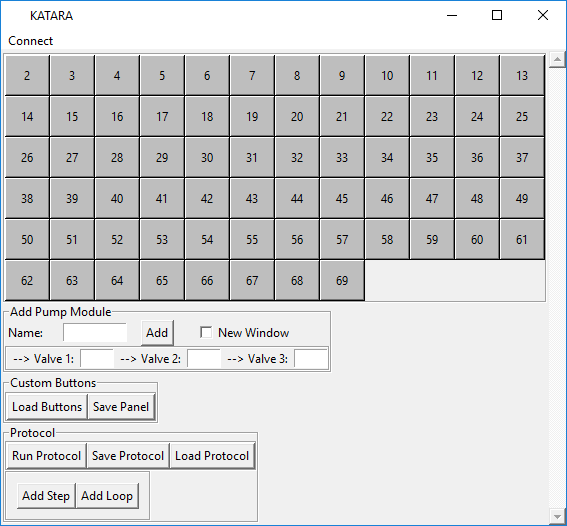


Figure S1. The main KATARA GUI window on a Windows computer.

Next, connect to the Arduino by selecting its Com port from the connect drop down menu (Figure S2). The Arduino must be plugged in before opening the connect menu.


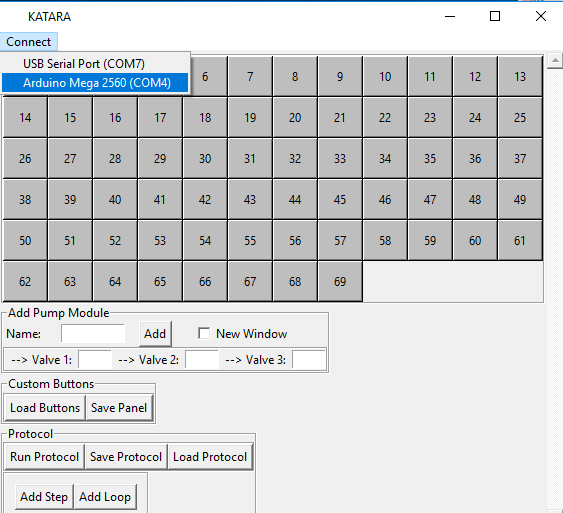


Figure S2. Connecting to an Arduino with the KATARA GUI.

After connecting, the GUI is ready to control valves. Use the panel of numbered buttons at the top of the window to open and close individual valves. When the Arduino sets a pin high, its button turns green when running in Linux or Windows (Figure S3). Button colors do not change on macs.


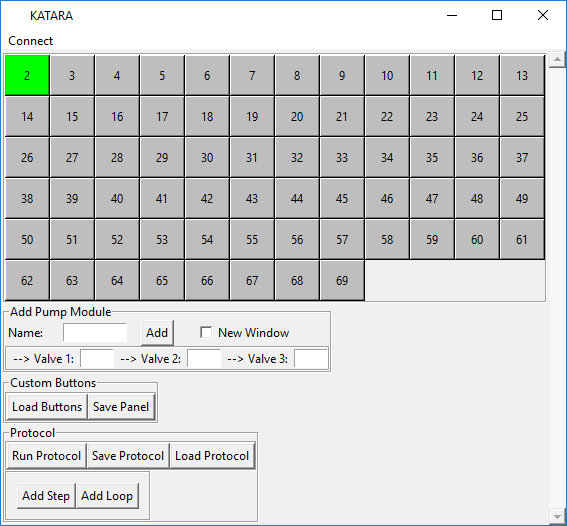


Figure S3. The “2” button is green indicating that pin 2 on the Arduino is set high.

To create an interface to control peristaltic pumps, enter the name of the pump in the name entry bar of the “Add Pump Module” interface, enter the valves that compose the peristaltic pump in the valve entry bars, then click “Add” (Figure S4). To open a pump interface in its own window, check the “New Window” box before clicking “Add.”


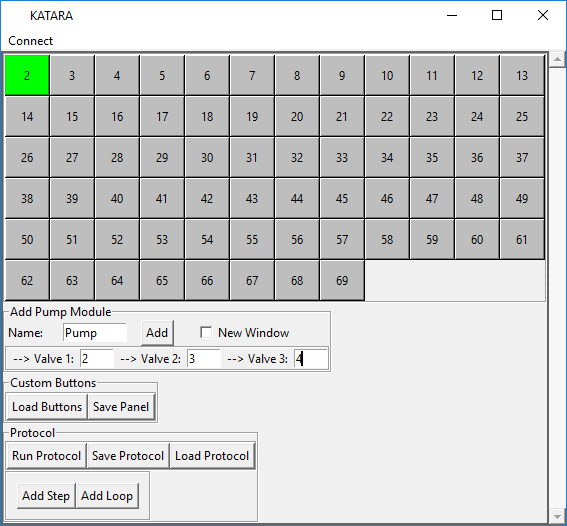


Figure S4. To add a peristaltic pump interface to the KATARA GUI, fill out the “Add Pump Module” box, and click the “Add” button.

After clicking, the “Add” button, a pump interface will appear below it (Figure S5). Users may add as many pump interfaces as desired.


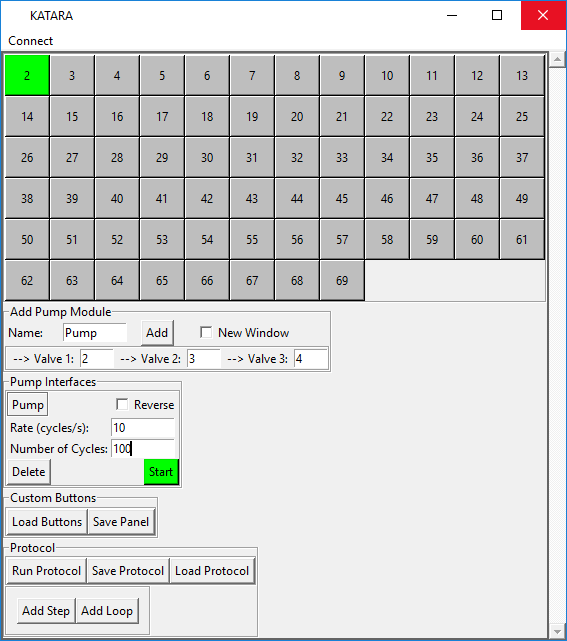


Figure S5. Pump interfaces allow users to specify the number of cycles, rate, and direction of a peristaltic pumping sequence.

The interface allows users to adjust the cycle frequency, number of cycles, and direction of pumping. While a pump is running, the buttons for its constituent valves will turn blue on Windows and Linux systems (Figure S6). The color will not change on macs. To cancel a pump sequence, press its “Stop” button (Figure S6), start another pump module, or actuate any valve on the valve button array. Valves used in peristaltic pumping sequences are left de-energized after pumping ends by default.


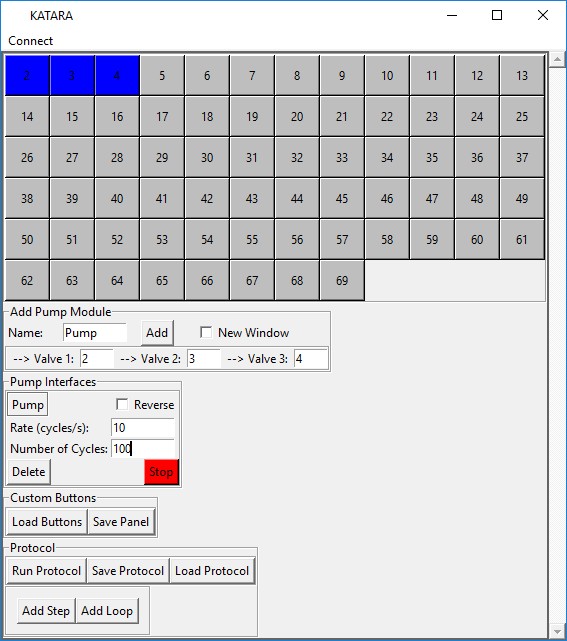


Figure S6. While a pump is running, its member valves turn blue.


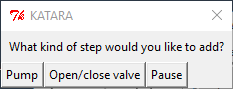


Figure S7. When adding a step, users can choose between pumping, valve open/closing or pausing.


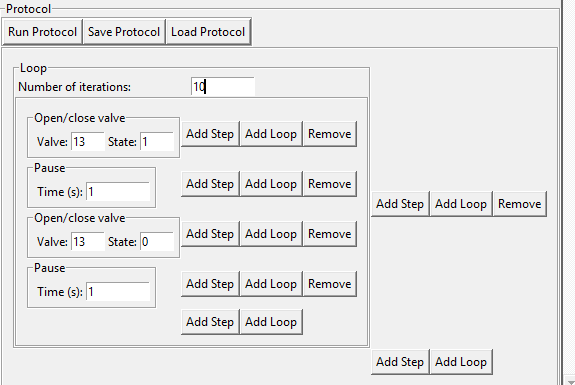


Figure S8. An example of a protocol that sets pin 13 high then low ten times.

The user can edit more complex sequences of peristaltic pumping and valve actuation in the protocol panel by adding pump steps, valve opening/closing steps, pause steps, and loops to repeat sequences. Clicking the “Add Step” button will open a window asking what kind of step to add (Figure S7). Figure S8 shows an example of a protocol that sets pin 13 high then low ten times. To run a protocol, press the “Run Protocol” button. When valves are energized or participate in peristaltic pump sequences, their buttons will turn green or blue respectively and the step that the protocol is on will turn green (Figure S9). To cancel a running protocol, press “Cancel Run” (Figure S9).


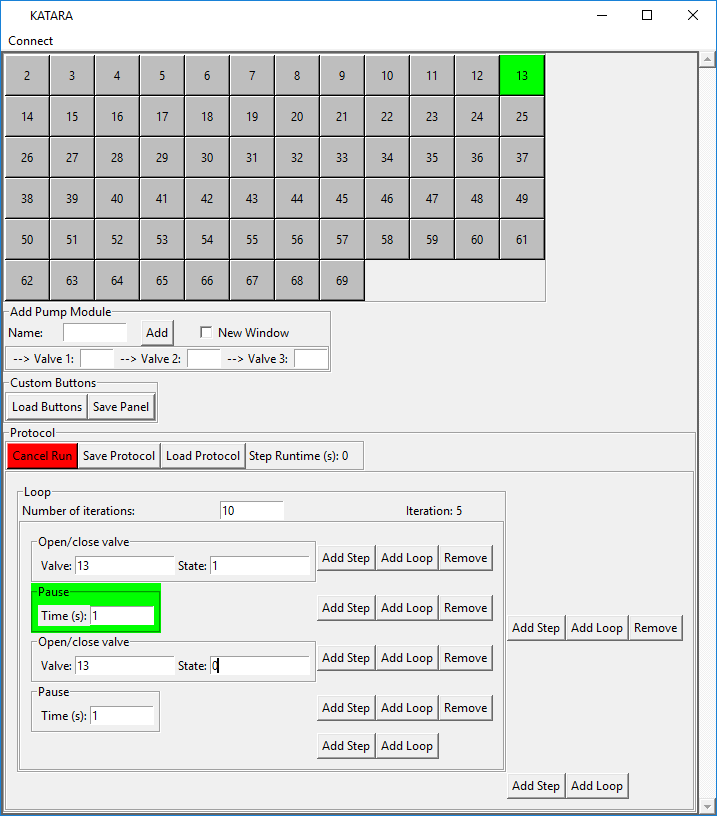


Figure S9. Running protocols display the changes they make to the valve configuration by changing valve button colors.

Valve steps can switch multiple pins at once. If switching many pins to the same state, enter the pins to switch as a comma-separated list, then the state (Figure S10). If switching valves to different states, include a corresponding comma separated list of states each valve should switch to (Figure S11). Switching many valves in a single step is faster than switching them in many steps because it allows the GUI to send a single serial command instead of many. Pump steps accept the number of cycles, pumping rate, and member valves as parameters (Figure S12).


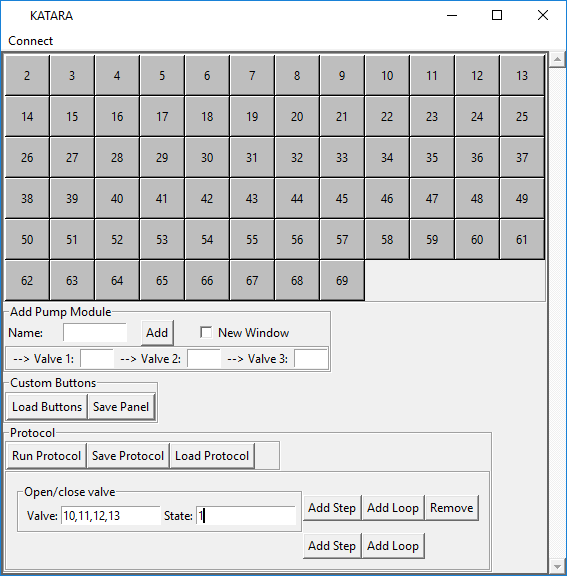


Figure S10. Valve steps can switch multiple pins high or low at once.


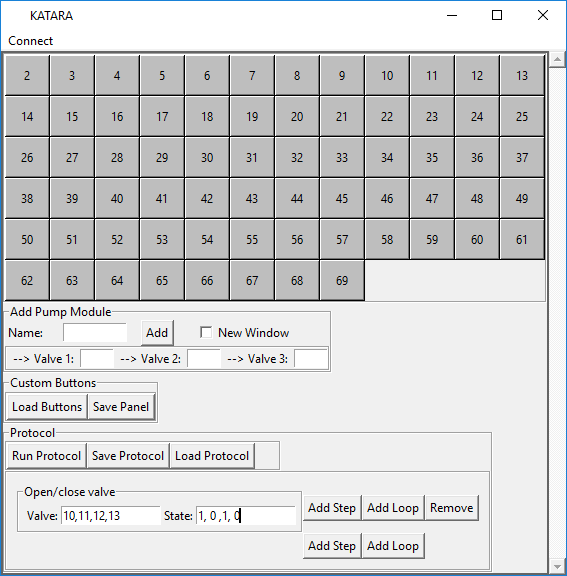


Figure S11. Valve steps can switch many valves to different states at once.


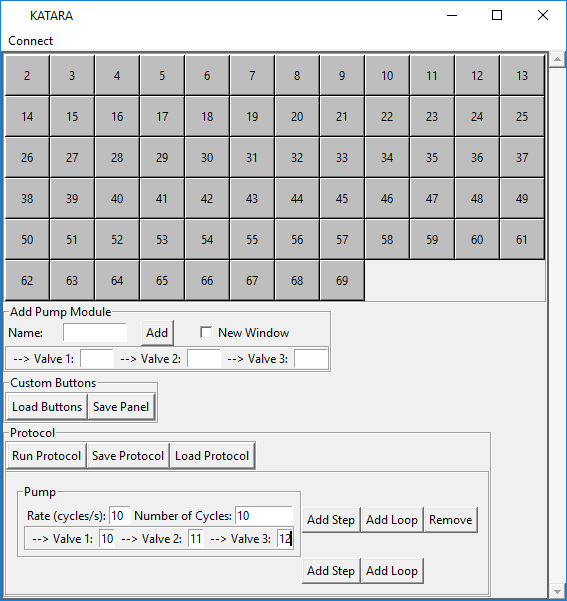


Figure S12. Pump steps accept pumping rates, number of pump cycles, and member valves as parameters.

Steps inside loops can accept python expressions involving the current loop iteration for their entry parameters. Loop iterations are stored in a python tuple, i, where the 0th entry of i, i[0], is the iteration of the current loop; the 1st entry, i[1], is the current iteration of the first outer loop; and so on. Loop iterations start at 1, and the iteration being executed is displayed in the upper right corner of the loop box when running (Figure S13). Figure S13 shows an example of a nested loop that iterates to sequentially set pins 2-11 and 12-21 high then low.


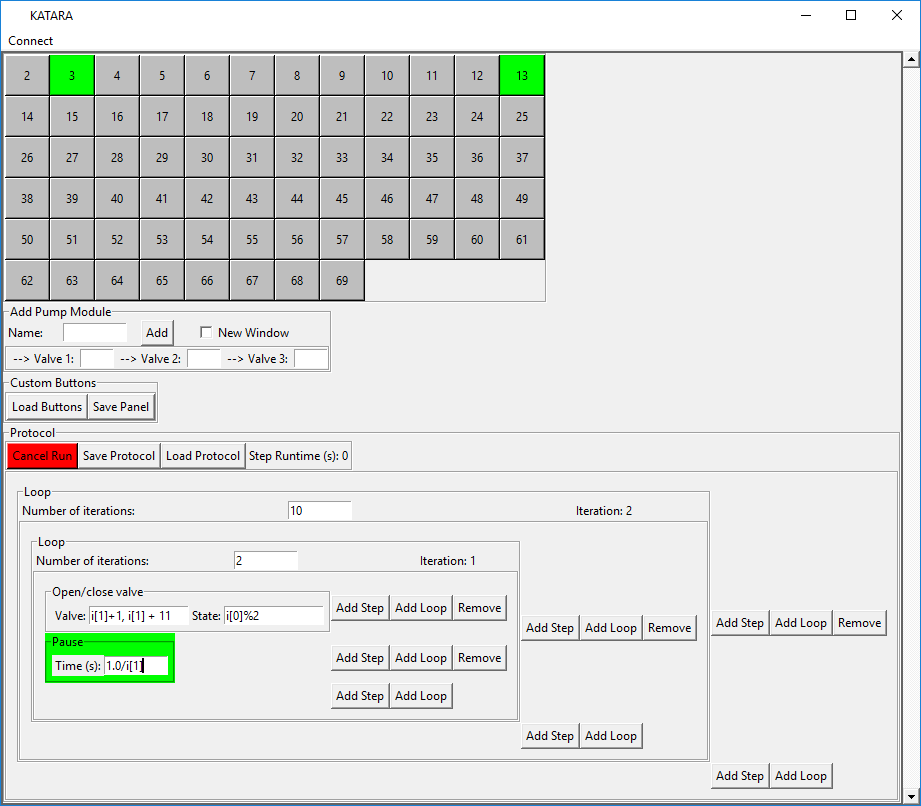


Figure S13. Steps inside nested loops can reference the iteration of their nth outer loop with i[n]. This figure shows a protocol that sequentially sets pins 2-11 and 12-21 high then low at an accelerating rate.

Users may save a protocol by clicking the “Save Protocol” button. The “Load Protocol” button opens a file dialogue window allowing users to locate saved protocols to load into the “Protocol” interface. Similarly, the “Load Buttons” button allows users to select saved protocols that may be called by pressing a button in the “Custom Buttons” panel (Figure S14). Custom button panels may be saved using the “Save Panel” button to be reloaded with the “Load Buttons” button at the start of future sessions.


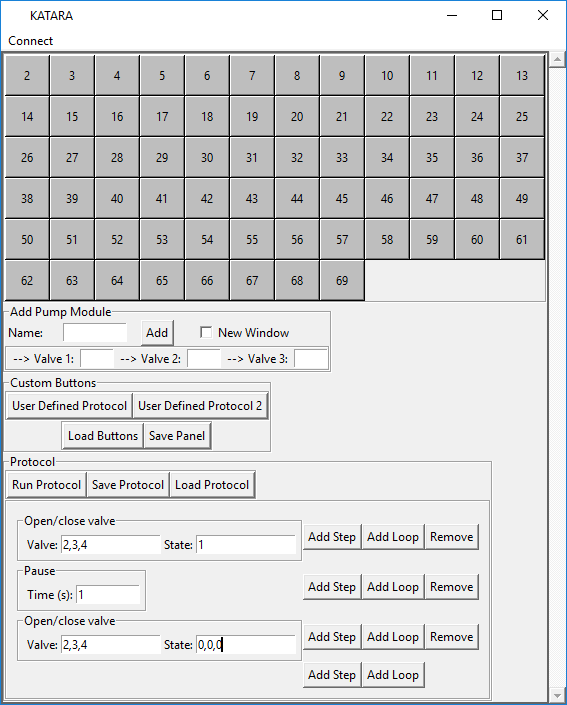


Figures S14. Saved protocols can be loaded as buttons in the “Custom Buttons” panel. A panel of custom buttons can be saved and reloaded at the start of later sessions.

**3. KATARA Programming Documentation**

The KATARA package consists of two classes for communicating with Arduinos running the KATARA Firmware: the KATARAValveController and the KATARAPump. Both are implemented in KATARAValveController.py. These classes manage serial communications for actuating individual valves and peristaltic pumping sequences in the KATARA GUI. Users should first create a KATARAValveController object to establish a connection to the Arduino, then use the KATARAValveController.specifyPump method to create auxiliary KATARAPump objects to control peristaltic pumps.

**Class** KATARAValveController - The KATARAValveController class establishes a serial connection to Arduinos running the KATARA firmware and provides methods to control the output state of digital pins.

*Constructor* KATARAValveController(str port) - *The KATARAValveController constructor establishes a serial connection to an Arduino running the KATARA firmware. It throws an error if it is unable to establish a connection.*

*Input:*

*port - string name of the serial port to open.*

*Output:*

*Returns a KATARAValveController object.*

*Method* isOpen() - *returns a boolean indicating whether the KATARAValveController object has an open connection to an Arduino running the KATARA firmware.*

*Input: None*

*Output: returns True if a connection is open, False if not.*

*Method* getPinState(int pin) - returns the current state of a pin on the Arduino, or throws an error if the pin argument is not an integer from 2 to 69.

*Inputs:*

*pin - the pin to inquire the state of: must be an integer from 2 to 69.*

*Output:*

*The state of the pin: 1 for high, 0 for low.*

*Method* setPins(tuple pins, tuple states) - *sets a tuple, list, or set of pins to the corresponding state in the corresponding tuple or list of states.*

*Inputs:*

*pins - a tuple, list, or set of pins. It must be the same length as the states tuple or list, have integer entries from 2 to 69, and no duplicate entries.*

*states - a tuple or list of states, 0 (low)or 1 (high), to set the pins: must be the same length as pins.*

*Output: Returns the responding message from the device as a string.*

*Method* testConnection() - *tests whether a serial connection to the Arduino firmware has been successfully established, throws an IOError if not.*

*Inputs: None*

*Output: None*

*Method* specifyPump(int v1, int v2, int v3) - *creates and returns a KATARApump object (see below) that can run peristaltic pump sequences using three valves.*

*Inputs:*

*v1 - the first valve in the peristaltic pump.*

*v2 - the second valve in the peristaltic pump.*

*v3 - the third valve in the peristaltic pump.*

*Output:*

*returns a peristaltic pump object for running peristaltic pump sequences (see below).*

**Class** KATARAPump - instances of this class are returned by the KATARAValveController.specifyPump method (see above). KATARAPump objects send serial commands to Arduinos running the KATARA firmware instructing them to run peristaltic pump sequences. If the Arduino receives a serial command while running a peristaltic pump sequence before it finishes, it will abort the pump sequence.

*Method* forward(int rate, int cycles, bool wait = False) - *runs the peristaltic pump in a forward sequence. Peristaltic pumping aborts if the Arduino receives a serial command before finishing.*

*Inputs:*

*rate - the rate at which to execute pump cycles (Hz).*

*cycles - the number of cycles to pump. Input -1 to run indefinitely until interrupted by a serial signal.*

*wait - boolean whether to pause the thread until the pumping sequence is finished.*

*Output: None*

*Method* reverse(int rate, int cycles, bool wait = False) - *runs the peristaltic pump in a reverse sequence. Peristaltic pumping aborts if the Arduino receives a serial command before finishing.*

*Inputs:*

*rate - the rate at which to execute pump cycles (Hz).*

*cycles - the number of cycles to pump. Input -1 to run indefinitely until interrupted by a serial signal.*

*wait - boolean whether to pause the thread until the pumping sequence is finished.*

*Output: None*

*Method* stop() - *sends a serial signal to stop a pumping sequence before completing all indicated cycles.*

*Input: None*

*Output: None*

**4. Extending the KATARA API and GUI to interface with valve control devices other than the KATARA Shield**

To extend the KATARA API and GUI to communicate with valve controllers other than the KATARA shield, write derived classes for ValveController, peristalticPump, and KATARAGUI. Overwrite methods as described below, and see the KATARAValveController and KATARAPump source code as example implementations.

**Class** ValveController

*Constructor* __init__(str port) - *derived methods must call ValveController.__init__ which initializes the member dictionary pinStates and establishes a serial connection to the device using the pyserial package. Derived methods must fill the pinStates dictionary with entries where the keys are the names of the pins for the controller and the values are the initialized state of the pins: 0 or 1.*

*Input:*

*port - string name of the serial port to open.*

*Output: None, but may raise errors.*

*Method* setPins(tuple pins, tuple states) - *derived method should give send serial commands to the valve controller to change the state of a list or tuple of pins. I recommend overwriting ValveController.write (below) and using it to wrap the serial.write command and handle IOErrors.
 Inputs:
 pins - a tuple or list of pins to set.
 states - a tuple or list corresponding to the pins*

*tuple indicating the state to set each pin.*

*Outputs: optional indication of successful write.*

Method testConnection() - *derived methods should send serial signals querying whether it is the correct device and check for the expected response. If there is no response, or the device does not respond correctly, raise an IOError.*

*Input: None*

*Output: None, but may raise errors.*

Method _write(str out) *derived classes should implement ValveController.write to send serial commands to the device and handle write errors. If you do not wish to handle IOErrors, you may send serial commands directly in the ValveController.setPins, persistalticPump._runPump, and peristalticPump.stop derived methods.*

*Inputs:*

*out - string to send over serial.*

*Outputs: None, but may raise warning or errors if IOErrors are encountered.*

Method - _checkPin(int valve) : *the _checkPin method checks user input to verify the input references a valid pin. If you do not override this function, ValveController._checkPin checks to see if the value is a key in the ValveController.pinStates dictionary. If there is no such key, it raises an error with message: "Error: invalid pin." Overwrite this method if you would like to provide a more helpful error message.*

*Inputs:*

*pin - user input for what should be a reference to an available pin.*

*Output: None, but throws error if pin is invalid.*

**Class** peristalticPump

Method _runPump(int rate, int cycles, str direction, bool wait = False) - *sends serial commands to execute a pumping sequence.*

*Inputs:*

*rate - the rate at which to actuate pump cycles (Hz).*

*cycles - the number of cycles to pump. Input -1 to run indefinitely.*

*direction - the direction to pump. Should be either string 'f' for forward or string 'r' for reverse.*

*wait - boolean whether to pause the thread until the pumping sequence is finished.*

*Output: None*

Method - stop() *stops a peristaltic pumping sequence before completion.*

*# Input: None*

*# Output: None*

**Class** KATARAGUI- You must override three methods: the setDeviceType method which specifies the name of the ValveController derived class that will send serial communications, the drawButtonPanelDim method which specifies the dimensions of the button panel grid, and maxPinCondition which specifies when to stop drawing pins.

Method setDeviceType() - tells the KATARAGUI the name of derived ValveController class to use. Override this function to set the devicetype member to your derived ValveController class.

Input: None

Output: None

Example from KATARAGUI:

**def** setDeviceType(self):

self.devicetype = KATARAValveController

Method drawButtonPanelDim() *- calls the drawButtonPanel method with specified dimensions, buttons_across by buttons_down.*

*Inputs: None*

*Outputs: None*

*Example from KATARAGUI:*

**def** drawButtonPanelDim(self):

buttons_across = 12 buttons_down = 6

self.drawButtonPanel(buttons_across, buttons_down)

maxPinCondition(self, int pin_num) - *since the number of buttons you want to draw will not always fit in a neat rectangle, this method checks if the drawButtonPanel method (above) has drawn buttons for all available pins. If so, it returns false telling drawButtonPanel to stop drawing new buttons.*

*Inputs:*

*pin_num - an integer pin number that is next to be drawn.*

*Output:*

*boolean: True if pin_num is greater than the maximum available pin, False if within bounds.*

*Example from KATARAGUI*

**def** maxPinCondition(self, pin_num):

**return** pin_num > 69
